# Spotted Fever Group Rickettsiae in Questing Ticks, Central Spain

**DOI:** 10.3201/eid1907.130005

**Published:** 2013-07

**Authors:** Isabel G. Fernández de Mera, Francisco Ruiz-Fons, Gabriela de la Fuente, Atilio J. Mangold, Christian Gortázar, José de la Fuente

**Affiliations:** Instituto de Investigación en Recursos Cinegéticos (IREC)–CSIC-UCLM-JCCM, Ciudad Real, Spain (I.G. Fernández de Mera, F. Ruiz-Fons, G. de la Fuente, C. Gortázar, J. de la Fuente);; Universidad Complutense de Madrid, Madrid, Spain (I.G. Fernández de Mera);; Instituto Nacional de Tecnología Agropecuaria, Santa Fe, Argentina (A.J. Mangold);; Oklahoma State University, Stillwater, Oklahoma, USA (J. de la Fuente).

**Keywords:** ticks, rickettsia, zoonosis, epidemiology, wildlife, spotted fever group rickettsiae, Spain, vector-borne infections

**To the Editor:** The number of spotted fever group (SFG) rickettsiae that cause diseases in humans is rapidly increasing (*1*,*2*); infections have been described in ticks and humans in Spain (*3*,*4*). However, in Castilla-La Mancha, central Spain, where recreational parks and hunting estates are abundant and humans may be exposed to infected ticks, information on such infections is not available. Therefore, it is worthwhile to characterize *Rickettsia* spp. found in this area for epidemiologic studies and proper diagnosis of possible rickettsial diseases.

In this study, we obtained 148 questing adult ticks, representing the most abundant species in the area: 12 *Dermacentor marginatus*, 26 *Rhipicephalus* *bursa*, 41 *Rh. sanguineus*, 15 *Rh. turanicus*, 8 *Rh. pusillus,* 2 *Haemaphysalis punctata*, 11 *Hyalomma lusitanicum,* and 33 *Hyalomma marginatum* (*5*). The ticks were collected from the vegetation at natural sites surveyed in Castilla-La Mancha by blanket dragging with a cotton flannelette during fall 2009 and spring–summer 2010 (Figure, panel A) and classified (*5*). 

**Figure:**
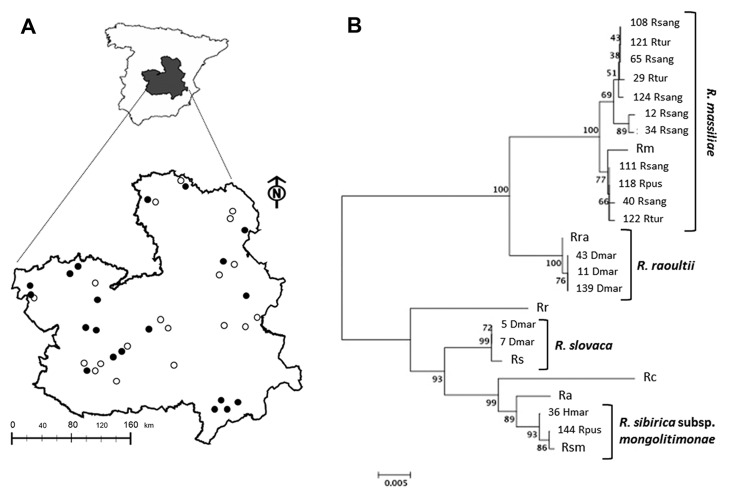
*Rickettsia* species in questing ticks collected in central Spain. A) Study area with 20 collection sites where ticks were found (black dots) of the 39 sites surveyed (white and black dots). B) Multilocus sequence analysis of *Rickettsia* spp. The evolutionary history was inferred by using the neighbor-joining method of *ompA*-*ompB* concatenated sequences (total length = 1,189 nt). The optimal tree with the sum of branch length = 0.15227017 is shown. The percentage of replicate trees in which the associated taxa clustered together in the bootstrap test (1,000 replicates) is shown next to the branches. The tree is drawn to scale, with branch lengths in the same units as those of the evolutionary distances used to infer the phylogenetic relationship. The evolutionary distances were computed using the Maximum Composite Likelihood method and are in the units of the number of base substitutions per site. Codon positions included were 1st+2nd+3rd+noncoding. All ambiguous positions were removed for each sequence pair. Evolutionary analyses were conducted in MEGA5 (www.megasofware.net). The number of the *Rickettsia* spp. recognized in this study is shown next to the tick spp. Identified with them. Clusters of identified *Rickettsia* spp. are shown. Rc, *Rickettsia conorii* strain Malish 7; Ra, *R. africae* strain ESF-5; Rr, *R. rickettsii* strain Iowa; Rs, *R. slovaca* strain 13-B; Rm, *R. massiliae* strain MTU5; Rsm, *R. sibirica* subsp. *mongolitimonae* strain HA-91; *R. raoultii* isolate XG86; Rhsang, *Rhipicephalus sanguineus*; Rtur, *Rh. turanicus*; Rpus, *Rh. pusillus*; Dmar, *Dermacenter marginatus*; Hmar, *Hyalomma marginatum.* Scale bar indicates number of nucleotide changes per site.

Total DNA was extracted from dissected tick internal organs by using the DNeasy Blood & Tissue Kit (QIAGEN, Düsseldorf, Germany) and used to analyze *Rickettsia* spp. DNA by PCR, cloning, and sequence analysis of the amplicons. At least 3 clones were sequenced for each amplicon. Genes targeted by PCR included fragments of adenosine triphosphate synthase α subunit (*atpA*), heat-shock protein 70 (*dnaK*), outer membrane protein A (*ompA*), outer membrane protein B (*ompB*), citrate synthase (*gltA*), 16S rRNA, *recA*, and initiator protein of DNA replication (*dnaA*) (*6*,*7*). To characterize *Rickettsia* spp., we compared nucleotide sequence identity to reference strains and carried out multilocus analysis using *ompA*-*ompB* sequences and in silico *PstI* and *Rsa*I restriction analysis of *ompA* sequences (*7*).

Ticks were first screened by 16S rRNA PCR, and positive samples were analyzed for all targeted genes. The results showed that 27 (18.2%) of the 148 ticks analyzed were positive for *Rickettsia* spp. Of these, 11 were confirmed as *R. massiliae* in *Rh. sanguineus*, *Rh. turanicus*, and *Rh. pusillus*, 3 as *R. raoultii* in *D. marginatus*, 2 as *R. slovaca* in *D. marginatus*, and 2 as *R. sibirica* subsp. *mongolitimonae* in *H. marginatum* and *Rh. pusillus* (Figure, panel B). These species had >99% pairwise nucleotide sequence identity to reference strains *R. massiliae* MTU5 (GenBank accession no. NC_009900), *R. slovaca* 13-B (accession no. NC_016639), and *R. sibirica* subsp. *mongolitimonae* HA-91 (accession no. AHZB00000000) genome sequences for all genes analyzed, and the only *R. raoultii* reported sequences (accession nos. JQ792107, JQ792166, JQ792134, and NR_043755 for *ompB*, *ompA*, *gltA*, and 16S rRNA, respectively). The sequences obtained in this study were deposited in the GenBank under accession nos. KC427998–KC428040.

Multilocus sequence analysis of *ompA*-*ompB* sequences (Figure, panel B) and in silico *PstI* and *Rsa*I restriction analysis of *ompA* sequences also confirmed the identity of the *Rickettsia* spp. identified in this study. As previously shown (*7*,*8*), multilocus analysis with *ompA*-*ompB* sequences was highly informative about the phylogenetic relationship between *Rickettsia* spp. (Figure, panel B), with similar results for maximum likelihood, maximum parsimony, and neighbor-joining methods (data not shown). Furthermore, the results suggested the tick vectors for these *Rickettsia* spp. in the study area (Figure, panel B) match those reported or suspected previously for these *Rickettsia* spp. (*1*–*4*), but for the first time, *R. sibirica* subsp. *mongolitimonae* was identified in *Hyalomma* and *Rhipicephalus* spp. ticks in Spain (*4*).

These tick species are frequently found in the same area feeding on Eurasian wild boar (*Sus scrofa*) and red deer (*Cervus elaphus*), which may act as hosts for these pathogens (*5*,*9*). To test this hypothesis, we determined the seroprevalence for SFG rickettsiae in these host species in Castilla-La Mancha. Serum samples from 235 red deer and 206 wild boar were analyzed for the presence of anti-SFG *Rickettsia* antibodies by ELISA (Spotted Fever Rickettsia IgG EIA Antibody Kit, Fuller Laboratories, Fullerton, CA, USA). The ELISA was adapted to test ungulate serum specimens by substituting antihuman IgG-horseradish by protein G-horseradish peroxidase (Sigma-Aldrich, Madrid, Spain). Specific SFG-*Rickettsia* antibodies were detected in 146 (70.9%) of 206 wild boar and 174 (74.0%) of 235 red deer, indicating a high seroprevalence in these species and thus the possibility that they can serve as hosts for these pathogens.

These tick species also infest humans, thus posing a risk for transmission of rickettsiae that are pathogenic in humans (*1*). In fact, Castilla-La Mancha is one of the regions in Spain where a high number of SFG rickettsioses are reported ([*10*]; http://pagina.jccm.es/sanidad/salud/epidemiologia/3507.pdf).

In conclusion, these results demonstrate that SFG rickettsiae with public health relevance are found in ticks in central Spain as in other regions in Spain. In central Spain, the widespread distribution of tick vectors and possible wildlife hosts, the presence of persons in tick-infested recreational and hunting areas, and the transstadial and transovarial transmission of the pathogen in ticks may favor transmission to humans.
